# A novel cuproptosis-related gene model predicts outcomes and treatment responses in pancreatic adenocarcinoma

**DOI:** 10.1186/s12885-023-10678-9

**Published:** 2023-03-10

**Authors:** Qixian Liu, Ruiyu Li, Huanwen Wu, Zhiyong Liang

**Affiliations:** Molecular Pathology Research Center, Department of Pathology, Peking Union Medical College Hospital, Chinese Academy of Medical Science & Peking Union Medical College, Beijing, China

**Keywords:** Pancreatic adenocarcinoma, Cuproptosis, Risk model, Tumor microenvironment, Prognosis, Treatment response, Immunotherapy

## Abstract

**Background:**

Cuproptosis is recently emerging as a hot spot in cancer research. However, its role in pancreatic adenocarcinoma (PAAD) has not yet been clarified. This study aimed to explore the prognostic and therapeutic implications of cuproptosis-related genes in PAAD.

**Methods:**

Two hundred thirteen PAAD samples from the International Cancer Genome Consortium (ICGC) were split into training and validation sets in the ratio of 7:3. The Cox regression analyses generated a prognostic model using the ICGC cohort for training (*n* = 152) and validation (*n* = 61). The model was externally tested on the Gene Expression Omnibus (GEO) (*n* = 80) and The Cancer Genome Atlas (TCGA) datasets (*n* = 176). The clinical characteristics, molecular mechanisms, immune landscape, and treatment responses in model-defined subgroups were explored. The expression of an independent prognostic gene *TSC22D2* was confirmed by public databases, real-time quantitative PCR (RT-qPCR), western blot (WB), and immunohistochemistry (IHC).

**Results:**

A prognostic model was established based on three cuproptosis-related genes (*TSC22D2*, *C6orf136*, *PRKDC*). Patients were stratified into high- and low-risk groups using the risk score based on this model. PAAD patients in the high-risk group had a worse prognosis. The risk score was statistically significantly correlated with most clinicopathological characteristics. The risk score based on this model was an independent predictor of overall survival (OS) (HR = 10.7, *p* < 0.001), and was utilized to create a scoring nomogram with excellent prognostic value. High-risk patients had a higher *TP53* mutation rate and a superior response to multiple targeted therapies and chemotherapeutic drugs, but might obtain fewer benefits from immunotherapy. Moreover, elevated *TSC22D2* expression was discovered to be an independent prognostic predictor for OS (*p* < 0.001). Data from public databases and our own experiments showed that *TSC22D2* expression was significantly higher in pancreatic cancer tissues/cells compared to normal tissues/cells.

**Conclusion:**

This novel model based on cuproptosis-related genes provided a robust biomarker for predicting the prognosis and treatment responses of PAAD. The potential roles and underlying mechanisms of *TSC22D2* in PAAD need further explored.

**Supplementary Information:**

The online version contains supplementary material available at 10.1186/s12885-023-10678-9.

## Introduction

Pancreatic adenocarcinoma (PAAD) has a 5-year survival rate of only approximately 10% [1, 2]. The poor prognosis is largely attributed to its high aggressiveness and resistance to existing therapies [3]. The conventional American Joint Committee on Cancer (AJCC) staging system is universally used to evaluate the patients’ risk and treatment requirements[4]. Nevertheless, this classification is limited in that it primarily focuses on clinical and pathological features, without considering molecular factors. Despite great progress in developing therapies (including chemotherapy, radiation therapy, immunotherapy, and targeted therapy) for PAAD, only a small number of patients can benefit from these treatments [5, 6]. Unfortunately, validated biomarkers to identify subgroups that may benefit from these treatments have not yet been developed [7]. Therefore, more accurate and effective biomarkers are urgently needed for identifying “high-risk” patients and those who would benefit from different interventions, optimizing clinical outcomes of PAAD.

Copper is a vital nutrient involved in many biological processes. Recently, copper dyshomeostasis was found to play an important role in cancer [8]. On the one hand, copper accumulation has been reported to promote tumor proliferation, growth, angiogenesis, and metastasis [9, 10], including in pancreatic neuroendocrine tumors [11]. On the other hand, with the worldwide success of platinum (II) compounds in cancer chemotherapy, copper complexes might also serve as potential antitumor agents [10]. However, the underlying mechanism remains largely unknown. Recently, a new form of programmed cell death pathway was discovered, named cuproptosis. Excess copper induced cuproptosis, which was distinct from all other known cell death forms, such as apoptosis, necrosis, ferroptosis, and autophagy [12]. In cuproptosis, excess copper could directly bind to lipoylated protein in the tricarboxylic acid (TCA) cycle, leading to proteotoxic stress and cell death. Clinical trials have shown that cuproptosis induced by copper ionophores helped cancer patients with low lactate dehydrogenase (LDH) respond better to treatment [13]. Cuproptosis has shown its therapeutic implications due to the preferential selectivity in cancer cells compared to normal cells [12]. Of note, accumulating studies have reported that cuproptosis-related genes are closely implicated in tumorigenesis, progression, prognosis, and drug sensitivity [14–16]. However, the role of cuproptosis-related genes in PAAD remains unclear.

This study aimed to develop a prognostic and therapeutic indicator for PAAD. 35 cuproptosis-related genes were screened using the Cox regression analyses to construct a prognostic model for PAAD. The model's accuracy was also validated with three other cohorts. We then investigated the correlations between the risk score based on the model and clinicopathological characteristics, and explored the independent prognostic role of the risk score in PAAD. A comprehensive comparative analysis of the molecular and immune landscapes, as well as the response of immunotherapy, targeted therapy, and chemotherapy between different risk groups, was also carried out. Besides, an independent prognostic predictor *TSC22D2* in PAAD was identified from the model, and its expression was verified by public databases and experiments. Our study provided an effective prognostic model based on cuproptosis-related genes and facilitated personalized therapy for PAAD patients.

## Materials and methods

### Data source

In total, transcriptomic information and clinical data of 472 PAAD patients are derived from three independent public datasets, including 213 samples in the International Cancer Genome Consortium (ICGC)-PACA-CA (https://dcc.icgc.org/releases/current/Projects), 79 samples (GSE85916) in Gene Expression Omnibus (GEO) (https://www.ncbi.nlm.nih.gov/geo/), and 176 samples in The Cancer Genome Atlas (TCGA)-PAAD cohort. The latest gene expression profiles and corresponding clinical follow-up information from the TCGA were downloaded through the UCSC Xena genome browser database (https://xenabrowser.net/datapages/) [17]. The download time of these datasets was April 14, 2022. Below were the steps involved in processing these data: 1) Samples without survival status, survival time, and clinical follow-up information was picked out from the analyses. 2) Genes with multiple gene symbols were averaged using the median expression. 3) Genes with Ensembl IDs were converted to gene symbols. 4) The gene expression data were transformed to the log2(TPM + 1) format.

213 samples of the ICGC-PACA-CA dataset were randomly divided into a training set (*n* = 152, 70%) and a validation set (*n* = 61, 30%). In case of the random division bias, the createDataPartition function of the “caret” R package was adopted to pre-randomize 213 samples 1000 times in advance. The division of the training set and validation set was unbiased because there is no difference in several features between the two sets, such as overall survival (OS), gender, age, AJCC stage, and histological grade (Supplementary Table 1). Besides, the testing set included samples from the TCGA-PAAD cohort and the GSE85916 cohort.

### Construction and validation of a prognostic model related to cuproptosis

A list of previously reported 35 cuprotosis-related genes was shown in Supplementary file 1 [12]. 34 cuproptosis-related genes were successfully matched with the training set. Univariate Cox regression analysis was used in the training set to explore the prognostic relevance of these genes for OS through the coxph function of the “survival” R package [18]. Then, a multivariate Cox regression analysis was conducted to obtain the coefficient of each gene for establishing the formula of the risk score. Patients were divided into two risk groups based on the median value of the risk score. Finally, the “timeROC” package was used in three additional datasets to analyze the specificity and sensitivity of 1-, 2-, and 3-year OS predictions. The area under the curve (AUC) was calculated to assess the ROC effect. The Kaplan–Meier (KM) curve analysis [19] was established by the “survminer” package to compare the OS between the high- and low-risk groups in the training, validation, and testing sets.

### Clinical correlation and prognostic values of the model

The correlations between the risk score and clinical characteristics were visualized by violin plot using the “ggplot2” package. Univariate and multivariate Cox regression analyses were used to evaluate the independent prognostic value of the 3-gene signature model and other clinical characteristics (tumor grade, T stage, N stage, gender, age, and AJCC stage). The results of univariate and multivariate Cox analyses based on the TCGA cohort were displayed as a forest plot by the “forestplot” package.

### Construction and validation of the nomogram model

The “rms” package was used to create a nomogram based on the independent prognostic factors in the multivariate Cox regression analysis [20]. Every variable in the nomogram scoring system was given a score, and the total score was calculated by adding all the scores together. A calibration curve was drawn to show the consistency of the nomogram prediction and clinical observation in one-, two-, and three-year OS.

### Analyses of the molecular and immune characteristics

“DESeq2” package was used to identify the differentially expressed genes (DEGs) between the high- and low-risk groups in the TCGA dataset. |Log2FoldChange|> 1 and a false discovery rate (FDR)-adjusted *P*-value < 0.01 were set as the cutoffs for the DEGs. Results were visualized in a volcano plot using the “ggplot2” package. Gene ontology (GO) functional enrichment analyses were performed on these DEGs with the “clusterProfiler” package [21]. Data on genetic alternations were downloaded from the TCGA data portal (https://portal.gdc.cancer.gov). The MutSig2.0 approach [22] was used to identify significantly mutated genes, and the top mutated genes in two risk groups were visualized by the “Maftools” package [23]. Correlation analysis was constructed between the risk score and total mutation burden (TMB). The differences in TMB and microsatellite instability (MSI) score between the two risk groups were also visualized by boxplot using the “ggplot2” package. Moreover, twenty-two subpopulations of tumor-infiltrating immune cells were analyzed using the CIBERSORT algorithm (https://cibersort.stanford.edu/) [24]. The different expressions of some immune checkpoints in the high- and low-risk groups were detected, such as CD274, CD276, CD44, and CD40.

### Prediction of treatment responses

In the immunotherapy response analyses, immunophenoscore (IPS) was a superior predictor of response to anti-cytotoxic T lymphocyte antigen-4 (CTLA-4) and anti-programmed cell death protein 1 (PD-1) antibodies [25]. IPS, available through The Cancer Immunome Atlas (TCIA) (https://tcia.at/), is developed from four categories: effector cells (activated CD4 + T cells, activated CD8 + T cells, effector memory CD4 + T cells, and effector memory CD8 + T cells), suppressive cells (Tregs and MDSCs), MHC-related molecules, and checkpoints or immunomodulators. Tumor Immune Dysfunction and Exclusion (TIDE) was calculated online (http://tide.dfci.harvard.edu/) and had potential clinical efficacy to assess the responsiveness of patients in different risk groups to immune checkpoint inhibitors (ICIs) therapy. The TIDE score is superior to recognized immunotherapy biomarkers (PD-L1 level, and interferon γ) for assessing anti-PD1 and anti-CTLA4 effectiveness. The responses to chemotherapy and target therapy were assessed using the “pRRophetic” package based on the Genomics of Drug Sensitivity in Cancer (GDSC) website (https://www.cancerrxgene.org/). A lower half-maximal inhibitory concentration (IC50) referred to a higher sensitivity to the drug treatment.

### Exploration of the expression and prognostic value of TSC22D2 in public datasets

GEPIA (http://gepia.cancer-pku.cn/) was used to investigate the mRNA expression of hub genes involved in the prognostic model in tumor samples (*n* = 179) and normal samples (*n* = 171). Patients were divided into two groups based on the median expression of the hub gene. The prognostic capabilities of these hub genes were respectively demonstrated using the KM curve in the TCGA, and ICGC datasets. To evaluate the expression of the hub gene at the protein level, we obtained the TCGA-PAAD proteomics cohort from the Clinical Proteomic Tumor Analysis Consortium (CPTAC), (https://proteomicscancer.gov/programs/cptac) including tumor samples (*n* = 137) and normal samples (*n* = 74).

### Validation of TSC22D2 RNA and protein expression in cell lines

Pancreatic cancer cell lines (AsPC-1 and BxPC-3) were cultured in RPMI-1640 (Corning, NY, USA) with 10% fetal bovine serum (FBS) and 1% penicillin–streptomycin. Two additional pancreatic cancer cell lines (PANC-1, MIA Paca-2) were cultured in DMEM (Dulbecco’ modified eagle medium) (Gibco, Grand Island, NY, USA) supplemented with 10% FBS and 1% penicillin–streptomycin. Human pancreatic ductal epithelium (hTERT-HPNE) cells were cultured in Medium D with mixtures of M3 and DMEM medium containing one volume of medium M3TM Base F culture media (InCell Corp., San Antonio, TX, USA), three volumes of glucose-free DMEM, 5% FBS, 5.5 mM glucose, 10 ng/ml EGF, and 50 µg/ml gentamycin [26]. All these cells were cultured at 37 °C in a humidified atmosphere containing 5% CO2. RNA was extracted from tissues using the TRIzol reagent (Invitrogen, Carlsbad, CA, USA) and was reverse-transcribed into cDNA using the PrimeScript RT Master Mix (Takara, Otsu, Shiga, Japan). RT-qPCR analyses were quantified with PowerUp™ SYBR® Green Master Mix (Applied Biosystems, Austin, TX, USA), and expression levels were normalized to *GAPDH* levels. Proteins were extracted in RIPA buffer supplemented with a complete, EDTA-free protease and phosphatase inhibitor single-use cocktail (Thermo Scientific). Proteins were separated by SDS-PAGE and blotted onto a PVDF membrane. Anti-*TSC22D2* (1:1000 dilution, #25,418–1-AP, Proteintech) was used as primary antibodies for immunoblotting. Reacted antibodies were detected using an enhanced chemiluminescence detection system.

### Statistical analysis

Categorical variables were analyzed using Fisher’s exact test or chi-square test. Continuous variables were analyzed by Wilcoxon rank-sum test or Kruskal–Wallis H test. Correlations between two continuous variables were examined by Spearman’s correlation analysis. All the statistical analyses were conducted by R software (version 4.1.3) and GraphPad Prism 8.0 (GraphPad Software Inc., San Diego, CA, The United States). All statistical tests were two-tailed unless otherwise stated. *P *< 0.05 were regarded as statistically significant.

## Results

### Constructing a prognostic model based on cuproptosis-related genes in the training set

**Fig. 1:**
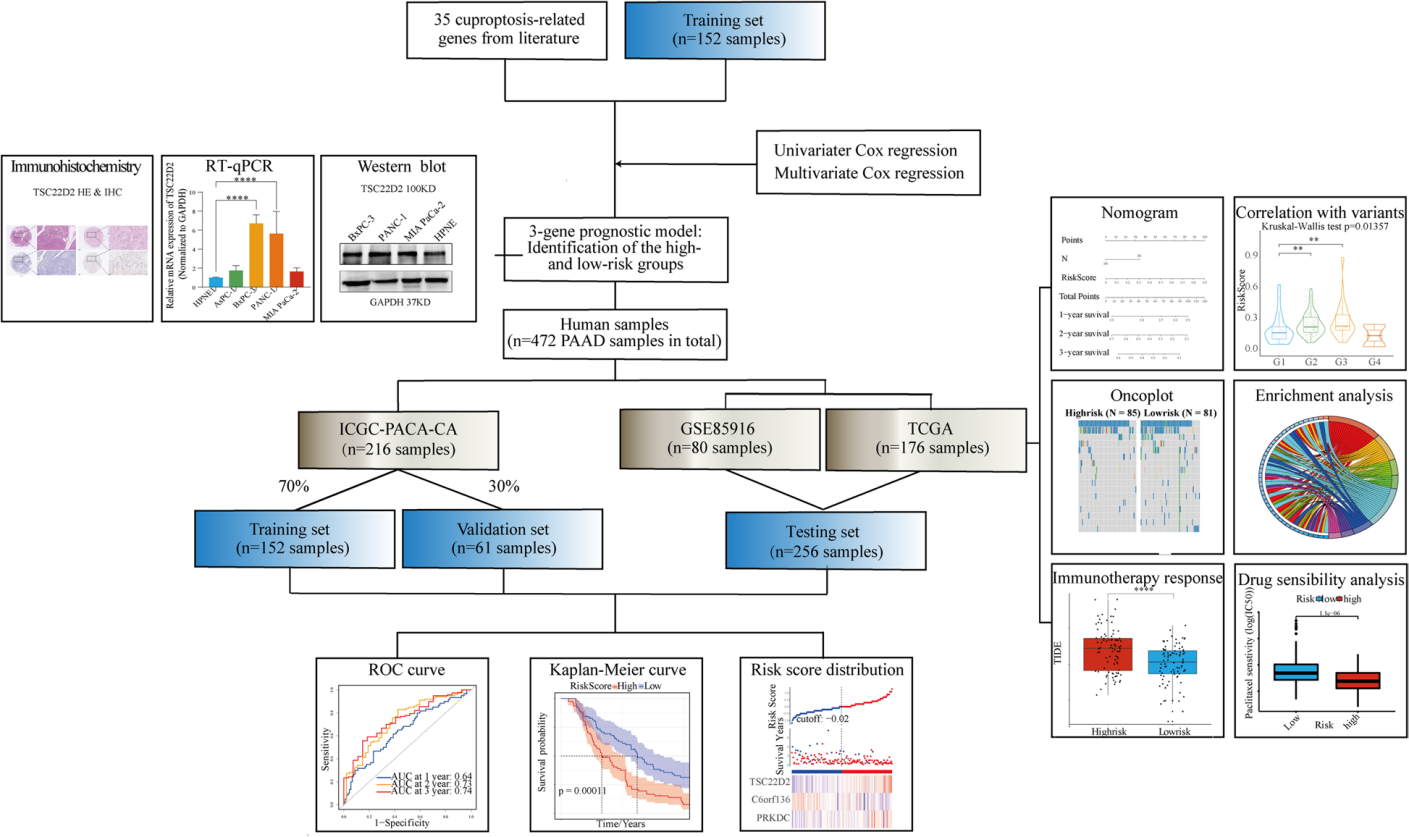
The overall study design and workflow

An overview of the main design of the present study was shown in Fig. 1. Among 34 cuproptosis-related genes, only three genes (*TSC22D2*, *C6orf136*, and PRKDC) were significantly associated with the OS of PAAD patients in the univariant Cox regression. The results of multivariate Cox regression were shown in Supplementary Fig. 1. It revealed that *PRKDC* (hazards ratios [HR] 1.34, 95%CI 1.10–1.63, *p* = 0.003), *C6orf136* (HR 0.71, 95%CI 0.54–0.92, *p* = 0.011), *TSC22D2* (HR 1.85, 95%CI 1.35–2.53, *p* < 0.001) contributed significantly to OS. Both *PRKDC* and *TSC22D2* were risk factors with HR > 1, whereas *C6orf136* was a protective factor with HR < 1. Then, a prognostic model was constructed for all samples using the formula based on the risk coefficients of these three genes:

$$Risk\;score\;=\;0.035\;\times\;e\hat{}\left(0.292\;\times\;PRKDC-0.347\;\times\;C6orf136\;+\;0.613\;\times\;TSC22D2\right)$$

**Fig. 2:**
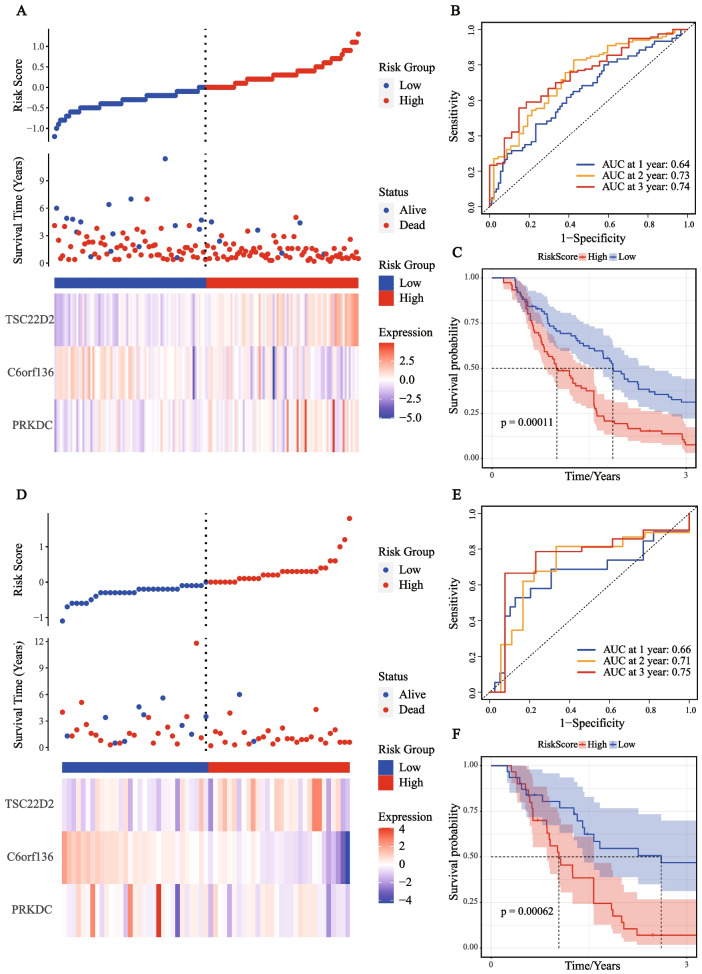
Construction and validation of the prognostic model in the training and validation sets. **A** The risk score, survival time, survival status, and 3-gene expression trend in the training set (ICGC, 152 samples). **B** ROC curves for the sensitivity and specificity of one-, two-, and three-year OS according to the risk score in the training set. **C** Kaplan–Meier curve for OS between the high- and low-risk groups in the training set. **D** The risk score, survival time, survival status, and 3-gene expression trend in the validation set (ICGC, 61 samples). **E** ROC curves for the sensitivity and specificity of one-, two-, and three-year OS according to the risk score in the validation set. **F** Kaplan–Meier curve for OS between the high- and low-risk groups in the validation set

Taking the median risk score as the cut-off value, 152 samples in the ICGC-training set were divided into the high-risk group (*n* = 76) and the low-risk group (*n* = 76). The distribution of the risk score showed that more death events were observed in the high-risk group (Fig. 2A). As the risk score increased, the expression of *TSC22D2* and *PRKDC* also elevated obviously, while the expression of *C6orf136* had a notable downward trend. Based on the ROC analysis, our model had a great prediction performance for OS in the training set (AUC of 1-year OS = 0.64; AUC of 2-year OS = 0.73; AUC of 3-year OS = 0.74, Fig. 2B). KM curve analysis in the training set revealed that patients in the high-risk group had a worse prognosis than those in the low-risk group (*p* = 0.00011, Fig. 2C).

### Evaluating the prognostic model in the validation set and testing set

All 61 samples in the ICGC-validation set were divided into the high-risk (*n* = 30) and the low-risk (*n* = 31) groups based on the median risk score using the risk formula. The risk score distribution was plotted in the validation set, which indicated that a higher risk score was associated with more death events (Fig. 2D). Subsequently, ROC curves were plotted, and the 1-year, 2-year, and 3-year AUCs of the OS prediction in the validation set were 0.66, 0.71, and 0.75, respectively (Fig. 2E). Then, KM curve analysis showed patients with a higher risk score tended to have a significantly worse prognosis than the low-risk group (*p* = 0.00062, Fig. 2F).

**Fig. 3:**
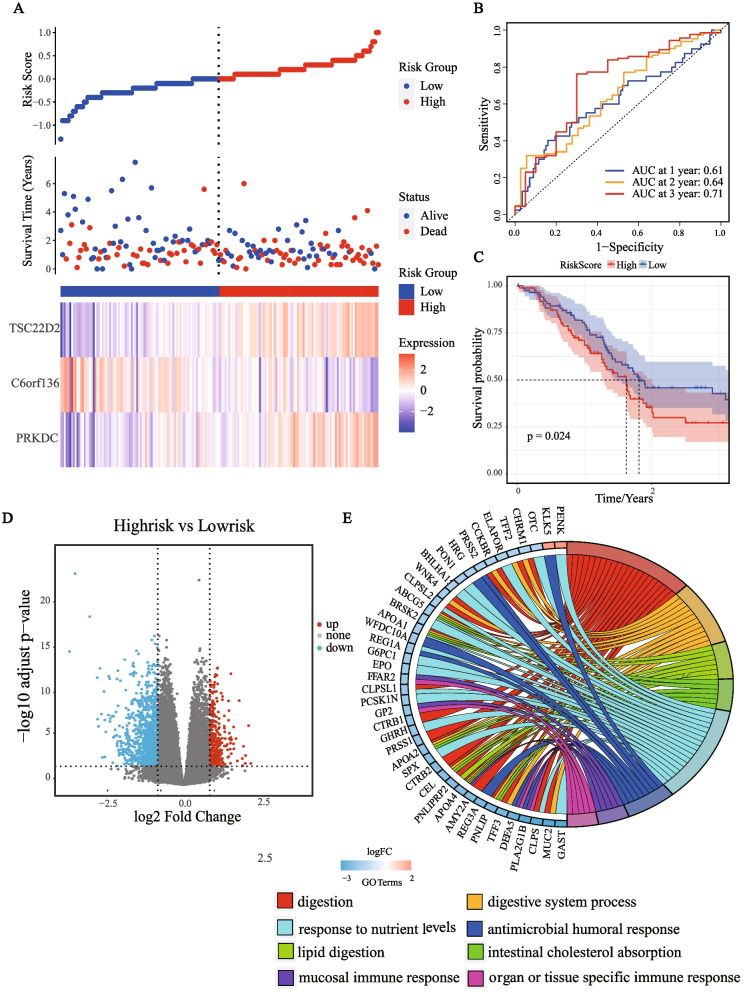
Evaluation of prognostic model in the TCGA-testing set and functional enrichment analysis of DEGs between the risk groups. **A** The risk score, survival time, survival status, and 3-gene expression trend in the testing set (TCGA, 176 samples). **B** ROC curves for the sensitivity and specificity of one-, two-, and three-year OS according to the risk score in the testing set. **C** Kaplan–Meier curve for OS between the high- and low-risk groups in the testing set. **D** Volcano plot of DEGs between the high-risk and low-risk groups from TCGA data set. **E** The Chord of eight significantly enriched GO terms between high-risk and low-risk groups

To further verify the robustness of our 3-gene prognostic model, 176 samples from the TCGA-PAAD dataset and 79 samples from the GSE85916 dataset were used as the testing set. The above analyses were also conducted separately in these two datasets. The distribution of the risk score also showed that the high-risk group tended to have a higher risk of death. The expression trends of three genes in the testing set (TCGA-PAAD cohort, Fig. 3A; GSE85916 cohort, Supplementary Fig. 2A) were consistent with that in the training and validation set. The 1-year, 2-year, and 3-year AUCs of this model in the TCGA-testing set were 0.61, 0.64, and 0.71, respectively (Fig. 3B). Besides, the 1-year, 2-year, and 3-year AUCs of the OS prediction in the GSE85916 testing set were 0.53, 0.62, and 0.68, respectively (Supplementary Fig. 2B). KM curve analysis revealed that the clinical outcomes of patients in the high-risk group were significantly worse (*p* = 0.024 in the TCGA-PAAD dataset, Fig. 3C; *p *= 0.05 in the GSE85916 dataset, Supplementary Fig. 2C).

To investigate the underlying mechanisms of the prognostic model, we performed a GO enrichment analysis of DEGs in the TCGA cohort. A total of 342 upregulated genes and 1149 downregulated genes were identified in the high-risk group (|log2FoldChange|> 1, *p* < 0.01), as shown in the volcano plot (Fig. 3D). A chord was plotted to show the functional enriched GO terms with *p*-value < 0.01 & FDR < 0.01 as the threshold (Fig. 3E). Interestingly, some immune response-related pathways were significantly enriched.

### Exploring the correlation between the risk score and clinicopathological features

**Fig. 4:**
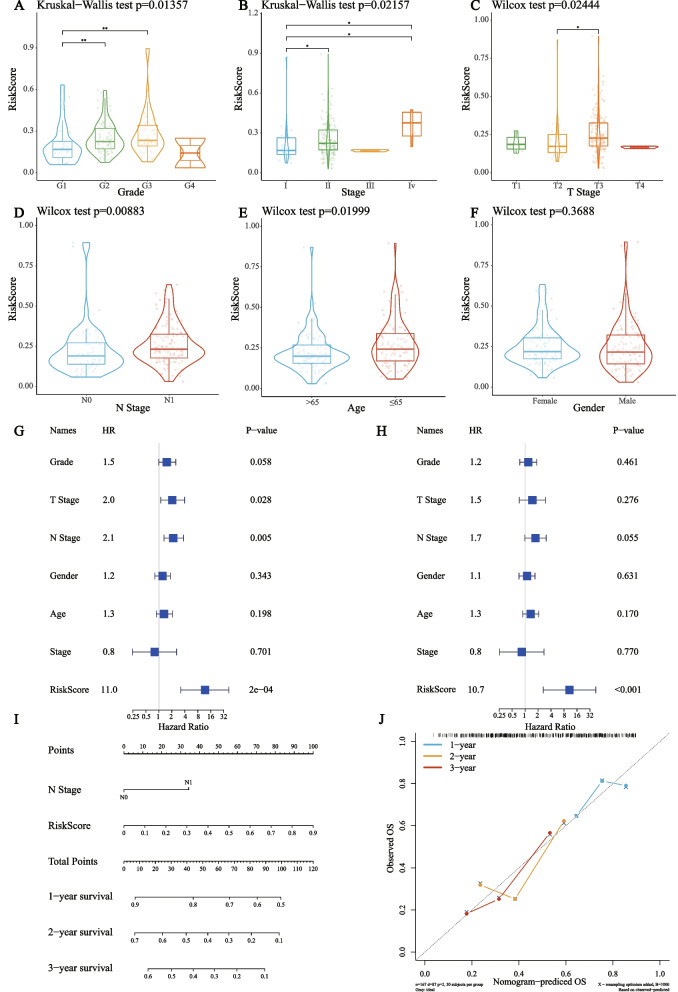
The clinical implications and prognostic role of the model. **A-F** Correlation between the risk score and various clinical characteristics. **G** Univariate Cox analysis of the risk score and clinical characteristics for OS in the TCGA cohort. **H** Multivariate Cox analysis of the risk score and clinical characteristics for OS in the TCGA cohort. **(I)** The nomogram built in combination with the risk score and the N stage predicted one-, two-, and three-year OS in the TCGA cohort. **J** The probabilities of OS at one-, two-, and three-year were assessed by the calibration curve of the nomogram in the TCGA cohort. * *p* < 0.05; ** *p* < 0.01; *** *P* < 0.001; **** *P* < 0.0001

The risk score was calculated in the TCGA cohort to examine its correlation with clinicopathological features (Fig. 4A-F). The risk score was significantly related to tumor grade, AJCC stage, T stage, and N stage (*p *< 0.05). patients with higher risk score tended to have a higher tumor grade and AJCC stage, a higher risk of lymphatic metastasis, and a larger tumor size. Patients under 65 years also had an increased risk score (*p *< 0.05). It is consistent with the previous cognition that younger PAAD patients may have a higher degree of tumor malignancy and a worse prognosis. Thus, our prognostic model provided a reliable prediction of clinicopathological characteristics.

### Assessing the independent prognostic value and prognostic accuracy of the model

To investigate whether the risk score based on the prognostic model was an independent prognostic factor, we performed univariate and multivariate Cox regression analyses in the TCGA-PAAD set. Univariate Cox regression analysis showed that T stage, N stage, and the risk score based on the prognostic model were significantly associated with OS (*p* < 0.05, Fig. 4G). Meanwhile, multivariate Cox regression analysis revealed that the risk score based on the prognostic model was the only independent prognostic indicator of OS (HR = 10.7, *p* < 0.001, Fig. 4H), which verified the robustness of our prognostic model again.

To make the prognostic model more clinically applicable, we constructed a nomogram model (Fig. 4I). The nomogram model was established using the N stage and the risk score since the risk score was highly significant (*p* < 0.001) and the N stage was marginally significant (*p* = 0.055) upon the multivariate Cox analysis. Both two elements were used to calculate each patient’s total score, predicting PAAD patients’ 1-year, 2-year, and 3-year survival probabilities. A calibration curve was plotted to analyze the consistency between the actual measured prognostic value and the prediction value by the nomogram. As shown in Fig. 4J, calibration curves of 1-year, 2-year, and 3-year OS in the TCGA cohort were near-optimal performance, which indicated that the prediction performance of the nomogram was great.

### Comparing the molecular characteristics and immune landscapes between the high- and low-risk groups

**Fig. 5:**
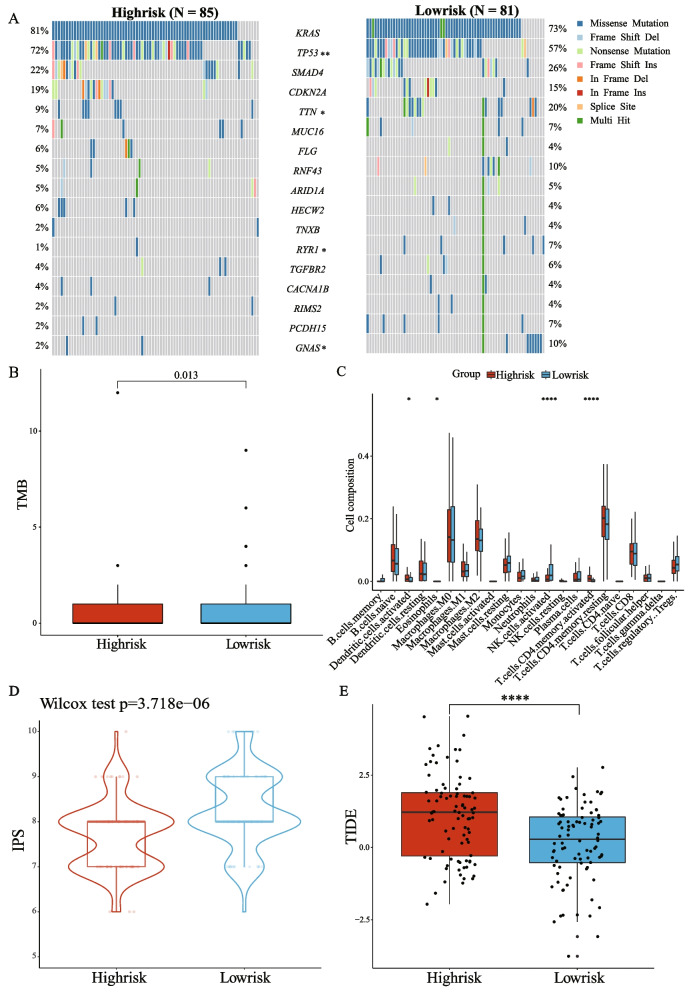
Comparing the molecular characteristics and immune landscapes between the high- and low-risk groups. **A** The oncoplot of somatic mutations in high- and low-risk patients using the TCGA cohort. **B** TMB between the high- and low-risk patients from the TCGA cohort. **C** Estimation of the proportions of immune infiltration cells using the CIBERSORT algorithm between the high- and low-risk groups. **D** The IPS score between the high- and low-risk patients from the TCGA cohort. **E** The TIDE score between the high- and low-risk patients from the TCGA cohort. * *p* < 0.05; ** *p* < 0.01; *** *P* < 0.001; **** *P* < 0.0001

The mutation profiles were analyzed and compared between the high- and low-risk groups. The top17 mutations in known driver genes of PAAD were displayed in the oncoplot (Fig. 5A). Missense mutations were the most common mutation type, followed by frameshift deletions and nonsense mutations. The mutation rates of the top four mutated genes (*KRAS*, *TP53*, *SMAD4*, and *CDKN2A*) were all higher than 15% in both groups. The mutation rate of the TP53 gene was significantly more common in the high-risk group (*p* < 0.01), while the mutation rate of *TTN*, *RYR1*, and *GNAS* genes was more common in the low-risk group (*p *< 0.05).

Subsequently, we explored the correlation of the risk score with TMB and MSI. As a result, the risk score was negatively correlated with TMB (R = -0.166, *p* < 0.05, Supplementary Fig. 3A). The high-risk group had a significantly lower TMB score (*p < *0.05, Fig. 5B), while there was no significant difference in MSI score between the two risk groups (Supplementary Fig. 3B).

As immune response-related pathways are associated with risk groups based on the model, we analyzed the distribution of 22 immune cells to compare the tumor microenvironment (TME) between two risk groups (Fig. 5C). Eosinophils and activated Natural killer (NK) cells were more abundant in the low-risk group, while activated dendritic cells and activated memory CD4 T cells were more plentiful in the high-risk group. Given the importance of ICIs in treating PAAD, we collected immunomodulatory genes for PAAD from previous literature and analyzed their expression in the two risk groups respectively (Supplementary Fig. 3C). As a result, almost all the immune checkpoint molecules were significantly higher in the high-risk group than in the low-risk group (*p* < 0.05).

### Predicting treatment responses based on risk groups

IPS was utilized to analyze the relationship between the risk groups and immune response. In the TCGA cohort, the low-risk group had a significantly higher IPS than that in the high-risk group (*p* < 0.001, Fig. 5D), indicating patients in the low-risk group may have a better response to ICIs. Besides, TIDE was also used to assess the potential clinical efficacy of immunotherapy in different risk groups. A higher TIDE score indicated a higher probability of tumor immune escape and thus inferior benefits from ICIs. Moreover, a higher TIDE score was related to a worse outcome. As shown in Fig. 5E, the high-risk group had a significantly higher TIDE prediction score than the low-risk group (*p* < 0.0001).

**Fig. 6:**
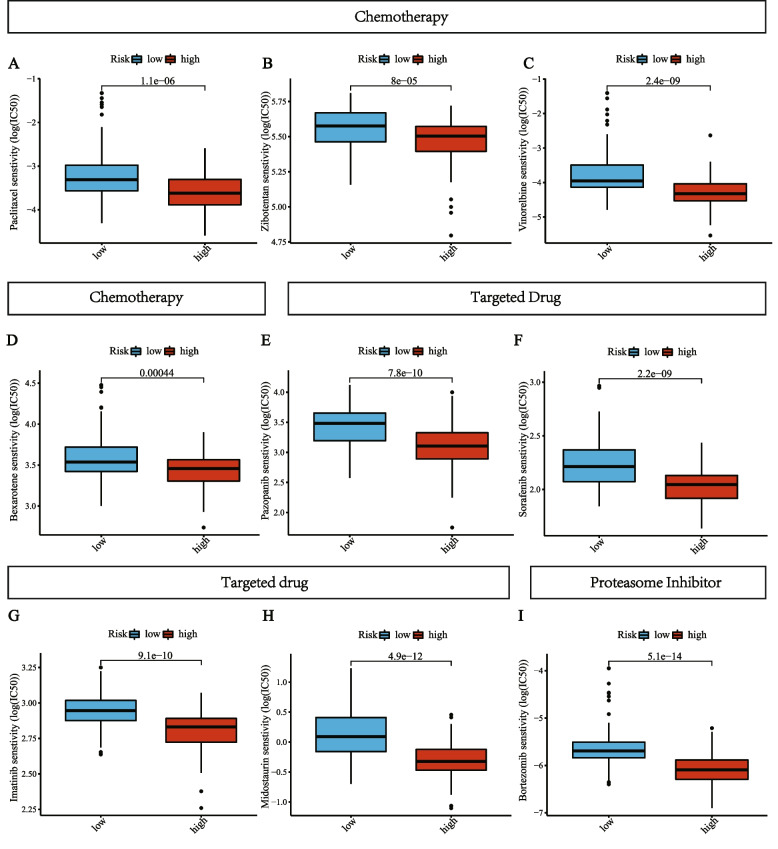
Association between the risk score and drug sensitivity, including chemotherapeutic and targeted agents, and a proteasome inhibitor. IC50: half-maximal inhibitory concentration

Chemotherapy and targeted therapy are important parts of multimodality treatment for pancreatic cancer [27]. However, due to the high resistance to chemotherapy and targeted therapy, many patients might not benefit from these treatments [28]. Thus, a total of 251 drugs from the GDSC website were estimated for chemotherapy and targeted therapy sensitivity in the high-risk and low-risk groups in the TCGA cohort. As shown in Fig. 6A-I, nine drugs had significantly different sensitivity between the two risk groups, including four chemotherapeutic agents (Paclitaxel, Zibotentan, Vinorelbine, Bexarotene), four targeted cancer drugs (Midostaurin, Pazopanib, Sorafenib, Imatinib), and a proteasome inhibitor (Bortezomib). The IC50 values of these chemotherapeutic agents and targeted therapies were significantly lower in the high-risk group (*p* < 0.001), indicating that patients in the high-risk group were more sensitive to these drugs.

### Identifying high expression of *TSC22D2* as a novel prognostic indicator in PAAD

**Fig. 7:**
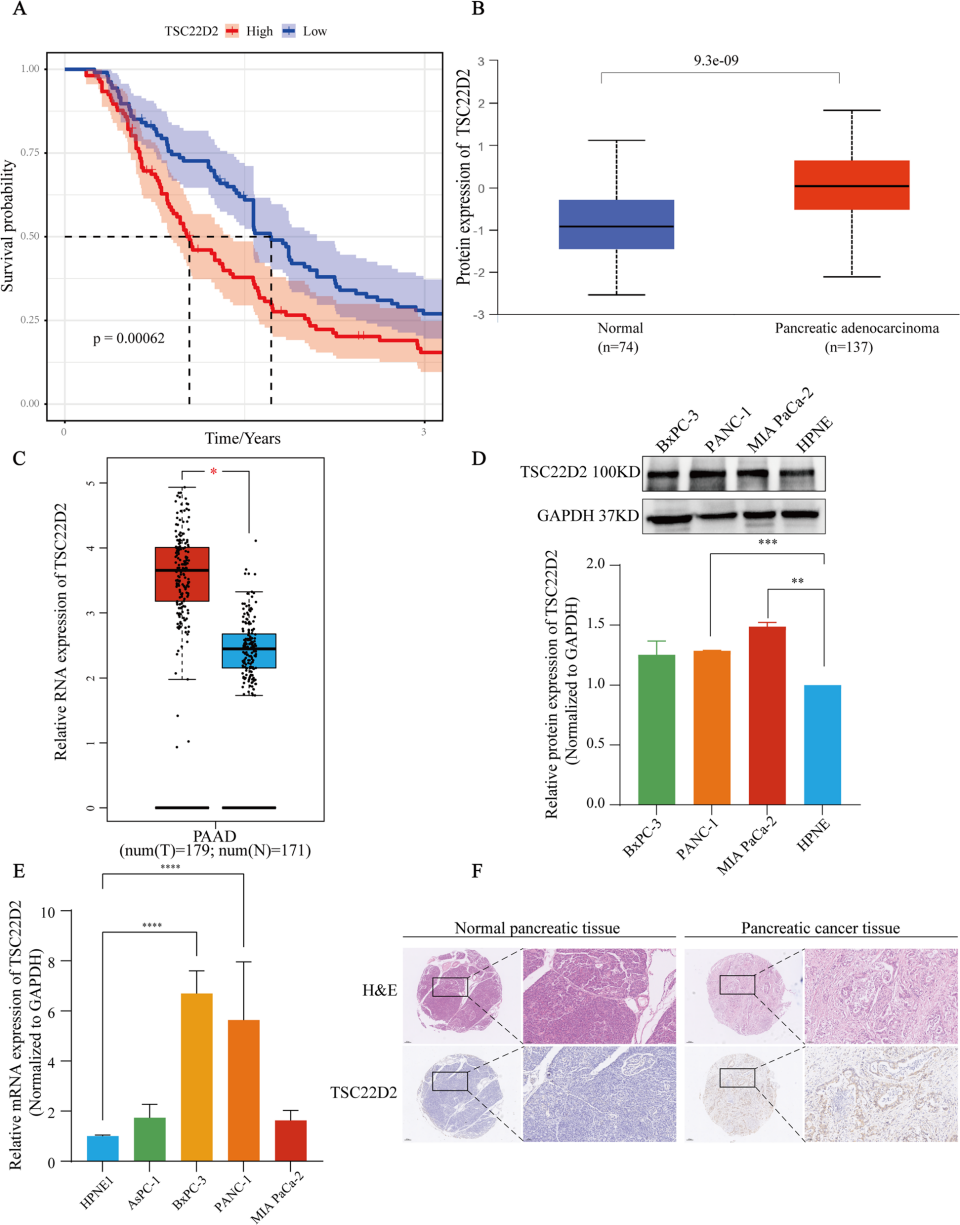
The prognostic value and expression of TSC22D2. **A** Kaplan–Meier curve for OS between the high- and low-*TSC22D2* expression group in the ICGC cohort.** B** CPTAC website showed the differences in *TSC22D2* expression between cancer tissues and normal tissues at the protein level. **C** Results from GEPIA indicated the relative mRNA expression of *TSC22D2* in PAAD samples and normal samples. **D** Bar chart shows quantification of *TSC22D2* protein levels compared to *GAPDH* control in three independent experiments. One representative blot is shown. Error bars show standard deviation. **E** Assessment of relative fold change in *TSC22D2* mRNA expression (HPNE set as 1) by RT-qPCR, normalized to *GAPDH*. Error bars show standard deviation* *p* < 0.05; ** *p* < 0.01; *** *P* < 0.001; **** *P* < 0.0001. **F** Hematoxylin and eosin (H&E) staining and IHC staining of *TSC22D2* was performed in pancreatic cancer tissues and normal pancreatic tissues. Scale bars: low magnification, 200 μm; high magnification, 100 μm

We screened each gene of our 3-gene prognostic model through KM curve analysis, respectively. Only *TSC22D2* was discovered to be significantly related to the prognosis of PAAD both in the ICGC and TCGA cohorts. High expression of *TSC22D2* was significantly associated with a worse prognosis in ICGC-PACA-CA samples (*p* < 0.001, Fig. 7A), which was similar in the TCGA-PAAD samples (*p* < 0.05, Supplementary Fig. 4A).

Next, data from CPTAC was obtained to investigate the expression of *TSC22D2* at the protein level. The protein expression of *TSC22D2* was significantly higher in PAAD samples (*n* = 137) than that in normal samples (*n* = 74) (*p* < 0.001), as shown in Fig. 7B. In addition, we analyzed the expression of *TSC22D2* at the RNA level. As shown in Fig. 7C, data from GEPIA showed that *TSC22D2* gene expression was significantly upregulated in pancreatic cancer tissues (*n* = 179) than that in normal tissues (*n* = 171) (*p* < 0.001).

Our western blot results revealed that pancreatic cancer cell lines (BxPC-3, PANC-1, MIA PaCa-2) had higher *TSC22D2* protein expression than hTERT-HPNE (Fig. 7D). *TSC22D2* protein level was 48.8% (*p < *0.01) higher in MIA PaCa-2 and 28.8% (*p* < 0.001) in PANC-1 than in control group (HPNE). The differential mRNA expression was also verified by RT-qPCR assays (Fig. 7E). The mRNA levels of *TSC22D2* were increased among all four pancreatic cancer cells as compared to HPNE control. Besides, BxPC-3 and PANC-1 have significantly higher expression levels than HPNE (*p* < 0.0001). The differential expression was consistent with the results from the Cancer Cell Line Encyclopedia (CCLE) database (Supplementary Fig. 4B). In addition, we detected the expression of *TSC22D2* in pancreatic cancer tissues and paired para-tumor normal tissues by immunohistochemistry (IHC) staining (Fig. 7F). The results showed that *TSC22D2* was significantly up-regulated in pancreatic cancer tissues compared with controls. Further explorations of its potential mechanism are currently investigating by us. Thus, an independent prognostic predictor of PAAD is *TSC22D2* high expression, and *TSC22D2* expression was also higher in pancreatic cancer tissues/cells than in normal tissues/cells.

## Discussion

PAAD is a highly malignant cancer with a very dismal prognosis [29]. The development of immunotherapy, targeted therapy, and chemotherapy for PAAD has been boosted, as molecular biology and immunology advanced [30–32]. A personalized treatment was urgently needed to maximize the effectiveness of diverse treatment options [33]. After decades of research on biomarkers for PAAD, reliable biomarkers for identifying “high-risk” PAAD patients and those who would benefit from treatments are still lacking [34, 35]. As a new type of cell death, cuproptosis may provide new insights for biomarker development of PAAD [12, 13, 16]. In our study, the Cox regression analyses were applied to establish a 3-gene prognostic model, which was an independent prognostic factor for OS. We then explored the clinical implications of the prognostic model and analyzed its predictive ability for various treatment responses, including immunotherapy, chemotherapy, and target therapy. In addition, high expression of *TSC22D2* was discovered to be an independent predictor of the poor prognosis of PAAD. The higher expression of *TSC22D2* in the pancreatic cancer tissues/cells was also confirmed by the public databases and experiments.

Referring to other signature-based studies, we used the median value of risk score as a cutoff for dividing patients into the high- and low-risk groups [34, 35]. Our model was verified as a valid and powerful prognostic biomarker for PAAD among three independent cohorts, with worse clinical outcomes in the high-risk patients and better survival in the low-risk patients. Our model's well-established prognostic significance led to the further investigation of its biological mechanisms. We revealed that DEGs between the risk groups were significantly enriched in several immune-related pathways. Gene mutations were compared between the two risk groups to gain further insight into the immunological nature of the two groups. Consistent with previous studies, missense mutations were the most common mutation type, followed by frameshift deletions and nonsense mutations. The most obvious difference in mutation profiles between the two risk groups was in *TP53* gene mutations, which were significantly more common in the high-risk group than in the low-risk group (*p* < 0.01). Besides, among four major driver genes for PAAD, the high-risk group had a higher frequency of mutation in *KRAS*, and *CDKN2A*. *KRAS* and *CDKN2A* mutation and alterations are early events in the development of pancreatic tumors [29]. *TP53*, the tumor suppressor gene, is mutated in over 70% of pancreatic ductal adenocarcinoma and is frequently linked to invasive and metastatic phenotype [36, 37]. It was proved that patients with *KRAS* or *TP53* mutation showed a worse prognosis than those without mutation (*p* = 0.0092 for *KRAS* and *p* = 0.013 for *TP53*) [38]. Interestingly, *KRAS*/*TP53* mutations were also demonstrated to be closely associated with immune escape in the mouse PDAC model KPC cells [39]. Therefore, there were relatively higher *TP53* mutations in the high-risk group, which might indicate their poorer prognosis and lesser sensitivity to ICIs.

Subsequently, we analyzed the relationship between the risk groups and known biomarkers for predicting immunotherapy responses, including TMB and MSI. TMB has also been regarded as a potential biomarker for predicting response to ICIs across many tumor types including PAAD [40]. High TMB leads to increased mutation-derived neoantigens that are likely to be recognized by the immune system, making tumors with high TMB more likely to respond to anti-PD-1/PD-L1 therapy [41]. According to our study, high-risk patients demonstrated relatively lower TMB, which might explain why high-risk patients were less likely to respond to ICIs and faced a greater risk of death. Additionally, it has been shown that immunotherapy is effective in rare MSI-high PAAD cases [42]. Thus, we also tested MSI scores in the high- and low-risk groups. However, the difference in MSI between the two risk groups was not significant. It was probably due to the very low prevalence of MSI in PAAD (1%-2%) [43]. The incidence of MSI was too low to detect a difference between the two risk groups.

The differences in TMEs between the two risk groups might also have important implications for immunotherapy. We revealed different compositions of immune cells between the two risk groups based on the prognostic model. The low-risk group had a higher proportion of activated NK cells and eosinophils than the high-risk group. As infection and tissue damage or inflammatory status occur, eosinophil migration, localization, and activation from the bone marrow were facilitated [44]. Besides, activated NK cells can mediate bone marrow rejection and promote engraftment, and elicit potent anti-tumor effects [45]. Thus, a higher proportion of eosinophils and activated NK cells was related to a better prognosis [44], which was consistent with our results that the low-risk group with more of these cells had a better outcome. In addition, we analyzed the expression of immune checkpoint molecules and found the majority of these molecules were upregulated in the high-risk patients, including PD-1/L1 and CTLA4. Hence, immune response suppression may be existed in the high-risk group of PAAD by "hijacking" the immune checkpoint pathway to achieve immune escape [46].

Both TIDE and IPS have been proven to be superior predictors of the treatment response to anti-CTLA-4 and anti-PD-1 antibodies [25, 47]. The IPS was developed by machine learning using the TCGA data to analyze tumor immunogenicity and tumor escape mechanisms. TIDE is a novel computational method to represent two mechanisms of tumor immune escape: the induction of T cell dysfunction in cytotoxic T lymphocytes (CTL)-high tumors and T cell exclusion in CTL-low tumors [47]. In our study, high-risk patients had higher TIDE and lower IPS scores than low-risk patients. It indicated that high-risk patients had higher levels of immune escape and might be less sensitive to ICI therapies. In addition, we also analyzed drug sensitivity between the high- and low-risk groups. The analyses of nine drugs revealed that patients in the high-risk group may receive benefits more from some chemotherapeutic agents and molecular-targeted drugs. Most of these drugs have been proven to be effective in treating PAAD patients during clinical trials. For example, nab-paclitaxel plus gemcitabine is a first-line treatment for advanced pancreatic cancer [48]. Sorafenib, an inhibitor of B-raf, VEGFR2, and PDGFR-β, has great activity against pancreatic cancer in a phase I trial [49]. Hence, our prognostic model may be used for optimizing individualized treatments for PAAD patients.

The prognostic model consisted of three cuproptosis-related genes (*PRKDC*, *C6orf136*, *TSC22D2*). Previous research identified that *TSC22D2* was a novel cancer-associated gene in a rare multi-cancer family [50]. In our study, high *TSC22D2* expression was found to act as an independent predictor for the poor prognosis of PAAD. High expression of *TSC22D2* was also related to a significantly higher AJCC tumor stage. However, in colorectal cancer, *TSC22D2* was considered as a tumor suppressor gene, and overexpressed *TSC22D2* inhibited tumor growth [51]. The expression of *TSC22D2* was reported to be significantly downregulated in colorectal cancer, while we found that the expression of *TSC22D2* was significantly higher in pancreatic cancer tissues/cells compared to the normal tissues/cells using public databases and experiments. Thus, *TSC22D2* plays a controversial role in cancers, especially in PAAD. Considering the pivotal role of *TSC22D2* in the process of cuproptosis, depletion of *TSC22D2* would reduce copper-induced cell death. We believe that *TSC22D2* may be utilized as a drug target for PAAD in the future, but the underlying mechanism still needs more research to explore. Additionally, *PRKDC* encodes DNA-dependent protein kinase catalytic subunit (DNA-PKcs), which is crucial for DNA double-strand break repair and V(D)J recombination [52]. Missense mutation of *PRKDC* results in deficient DNA-PKcs, which is associated with the inflammatory disease with organ-specific autoimmunity [53], as well as the loss of mature T and B cells and jak3 in T and putative Natural Killer cells [54]. *C6orf136* is a conserved gene, which is hypermethylated responding to oncogene *FOXM1* expression in head neck squamous cell carcinoma tissue cells [55]. Moreover, *C6orf136* was reported to be a core gene in a signature, which was identified as a novel biomarker for bladder cancer [56]. In the calculation formula of the risk score, the coefficients of *PRKDC* and *TSC22D2* were positive numbers, indicating a positive relationship between the risk score and *PRKDC* and *TSC22D2*. While *C6orf136* was negatively correlated with the risk score. In conclusion, the above studies supported that our prognostic model was a potential measurable prognostic biomarker for PAAD patients.

Although the prognostic model in PAAD is promising, some limitations should be mentioned. First, as a result of the limited sample size, selection bias may exist. Second, since the number of known cuproptosis-related genes is limited, the model exhibits only moderate efficacy. Third, as the data were mainly obtained from public databases, the actual predictive value of the model needs to be validated with laboratory/real clinical data from large prospective studies prior to clinical application. Finally, the precise roles and molecular mechanisms of cuproptosis-related genes including *TSC22D2* in PAAD have not yet been addressed through biological experiments.

In conclusion, we propose a novel model based on cuproptosis-related genes, which has great potential in predicting prognosis and therapy responses. The precise roles and underlying mechanisms of cuproptosis in PAAD need further explored.

## Supplementary Information

**Additional file 1. Supplementary File S1. **Cuproptosis-related genes selected by genome-wideCRISPR-Cas9 loss-of-function screens after treatment with elesclomol-copper inOVISE cells. **Supplementary Table 1.** Differences in clinicopathological characteristics betweenICGC training set and ICGC validation set. **Supplementary Figure 1.** The hazard ratio of three genes was calculated bymultivariate Cox regression analysis in the prognostic model. **Supplementary Figure 2.** Evaluation of prognostic model in the GEO testing set. (A)The risk score, survival time, survival status, and 3-gene expression trend in thetesting set (GSE85916, 80 samples). (B) ROC curves for the sensitivity andspecificity of one-, two-, and three-year OS according to the risk score in theGEO-testing set. (C) Kaplan–Meier curve for OS between the high- and low-riskgroups in the GEO-testing set. **Supplementary Figure 3.** Comparison of the molecular characteristics and immunelandscapes between the risk groups. (A) Correlation between the risk score and TMB.The Spearman correlation coefficients (R) and corresponding p values are shown. (B)The relationship between the risk score and MSI in TCGA-PAAD cohort. (C) Theexpression levels of representative immune checkpoint genes in the high- andlow-risk PAAD patients from the TCGA cohort. * *p* < 0.05; ** *p* < 0.01; *** *P* <0.001; **** *P* < 0.0001. **Supplementary Figure 4.** The prognostic value and expression of TSC22D2. (A)Kaplan–Meier curve for OS between the high- and low-TSC22D2 expression groupsin the TCGA cohort. (B) The expression of TSC22D2 in pancreatic cancer cells wasanalyzed in the Cancer Cell Line Encyclopedia database. **Supplementary Figure S1.** (A-B) Original western blot images of TSC22D2 andGAPDH.

## Data Availability

The datasets generated in this study can be found in The Cancer Genome Atlas (pancreatic adenocarcinoma cohort), International Cancer Genome Consortium (pancreatic adenocarcinoma cohort), and the Gene Expression Omnibus (GEO) (https://www.ncbi.nlm.nih.gov/geo/) under the accession numbers GSE85916.

## Abbreviations

PAAD: Pancreatic adenocarcinoma
ICGC: International Cancer Genome Consortium
GEO: Gene Expression Omnibus
TCGA: The Cancer Genome Atlas
RT-qPCR: Real-time quantitative PCR
WB: Western blot
OS: Overall survival
HR: Hazards ratios
AJCC: American Joint Committee on Cancer
TCA: Tricarboxylic acid
LDH: Low lactate dehydrogenase
AUC: Area under the curve
KM: Kaplan–Meier
DEG: Differentially expressed gene
FDR: False discovery rate
GO: Gene ontology
TMB: Total mutation burden
MSI: Microsatellite instability
IPS: Immunophenoscore
CTLA-4: Cytotoxic T lymphocyte antigen-4
PD-1: Programmed cell death protein 1
TCIA: The Cancer Immunome Atlas
TIDE: Tumor Immune Dysfunction and Exclusion
ICI: Immune checkpoint inhibitors
GDSC: Genomics of Drug Sensitivity in Cancer
IC50: Half-maximal inhibitory concentration
CPTAC: Clinical Proteomic Tumor Analysis Consortium
FBS: Fetal bovine serum
hTERT-HPNE: Human pancreatic ductal epithelium
CCLE: Cancer Cell Line Encyclopedia
CTL: Cytotoxic T lymphocytes
DNA-PKcs: DNA-dependent protein kinase catalytic subunit
