# Supplementary material for: A novel cuproptosis-related gene model predicts outcomes and treatment responses in pancreatic adenocarcinoma
Source: BMC Cancer. 2023 Mar 10;23:226. doi: 10.1186/s12885-023-10678-9 (PMC9999523; doi:10.1186/s12885-023-10678-9)

Supplementary File S1. Cuproptosis-related genes selected by genome-wide CRISPR-Cas9 loss-of-function screens after treatment with elesclomol-copper in OVISE cells.

Supplementary Table 1. Differences in clinicopathological characteristics between ICGC training set and ICGC validation set.

Supplementary Figure 1. The hazard ratio of three genes was calculated by multivariate Cox regression analysis in the prognostic model.

Supplementary Figure 2. Evaluation of prognostic model in the GEO testing set. **(A)** The risk score, survival time, survival status, and 3-gene expression trend in the testing set (GSE85916, 80 samples). **(B)** ROC curves for the sensitivity and specificity of one-, two-, and three-year OS according to the risk score in the GEO-testing set. **(C)** Kaplan–Meier curve for OS between the high- and low-risk groups in the GEO-testing set.

Supplementary Figure 3. Comparison of the molecular characteristics and immune landscapes between the risk groups. **(A)** Correlation between the risk score and TMB. The Spearman correlation coefficients (R) and corresponding p values are shown. **(B)** The relationship between the risk score and MSI in TCGA-PAAD cohort. **(C)** The expression levels of representative immune checkpoint genes in the high- and low-risk PAAD patients from the TCGA cohort. * $p < 0.05$; ** $p < 0.01$; *** $P < 0.001$; **** $P < 0.0001$.

Supplementary Figure 4. The prognostic value and expression of *TSC22D2*. **(A)** Kaplan－Meier curve for OS between the high- and low-*TSC22D2* expression groups in the TCGA cohort. **(B)** The expression of *TSC22D2* in pancreatic cancer cells was analyzed in the Cancer Cell Line Encyclopedia database.

Supplementary Figure S1. (A-B) Original western blot images of TSC22D2 and GAPDH.

Supplementary File S1

gene	FDR
CDKN2A	5.73E-04
OXA1L	5.73E-04
SOX2	5.73E-04
TKT	5.73E-04
AHR	5.73E-04
TMEM191E	5.73E-04
DLAT	5.73E-04
LIAS	5.73E-04
MBTPS1	5.73E-04
RPAP1	5.73E-04
MBTPS2	5.73E-04
LIPT1	5.73E-04
FDX1	5.73E-04
PDHB	5.73E-04
SCAP	5.73E-04
RPL3	0.00161213
PDHA1	0.002149427
CAPRIN1	0.002149427
LDLR	0.002149427
UBAP2L	0.002149427
IDH2	0.00273578
ZFAT	0.00273578
SAMM50	0.004112087
PITRM1	0.004298978
PGP	0.004298978
DLD	0.004298978
PRKDC	0.005040257
TSC22D2	0.005040257
CERT1	0.005040257
INTS6	0.00659179
TADA2A	0.006933843
MTF1	0.00703478
API5	0.00703478
C6orf136	0.007333622
GLS	0.008106686

Supplementary Table 1

**Variants**	**ALL**	**ICGC training set**	**ICGC validation set**	**P Value**
	**N=213 (percent of whole cohort)**	**N=152 (percent of ICGC training set)**	**N=61 (percent of ICGC validation set)**	
**Overall survival**				0.349
Alive	34 (16.0%)	22 (14.5%)	12 (19.7%)	
Death	179 (84.0%)	130 (85.5%)	49 (80.3%)	
**Gender**				0.916
Female	86 (40.3%)	62 (40.8%)	24 (39.3%)	
Male	136 (63.8%)	90 (59.2%)	36 (59.0%)	
**Age**				0.974
≥65 years	111 (52.1%)	80 (52.6%)	31 (50.8%)	
＜65 years	66 (31.0%)	47 (30.9%)	19 (31.1%)	
**AJCC stage**				0.745
Stage I/II	159 (74.6%)	114 (75%)	45 (73.8%)	
Stage III/IV	9 (4.2%)	6(3.9%)	3 (4.9%)	
**Histological grade**				0.606
Well	20 (9.4%)	13 (8.6%)	7 (11.5%)	
Moderate	57 (26.8%)	43 (28.3%)	14 (23.0%)	
Poor	49 (23.0%)	37 (24.3%)	12 (19.7%)	

Supplementary Figure 1

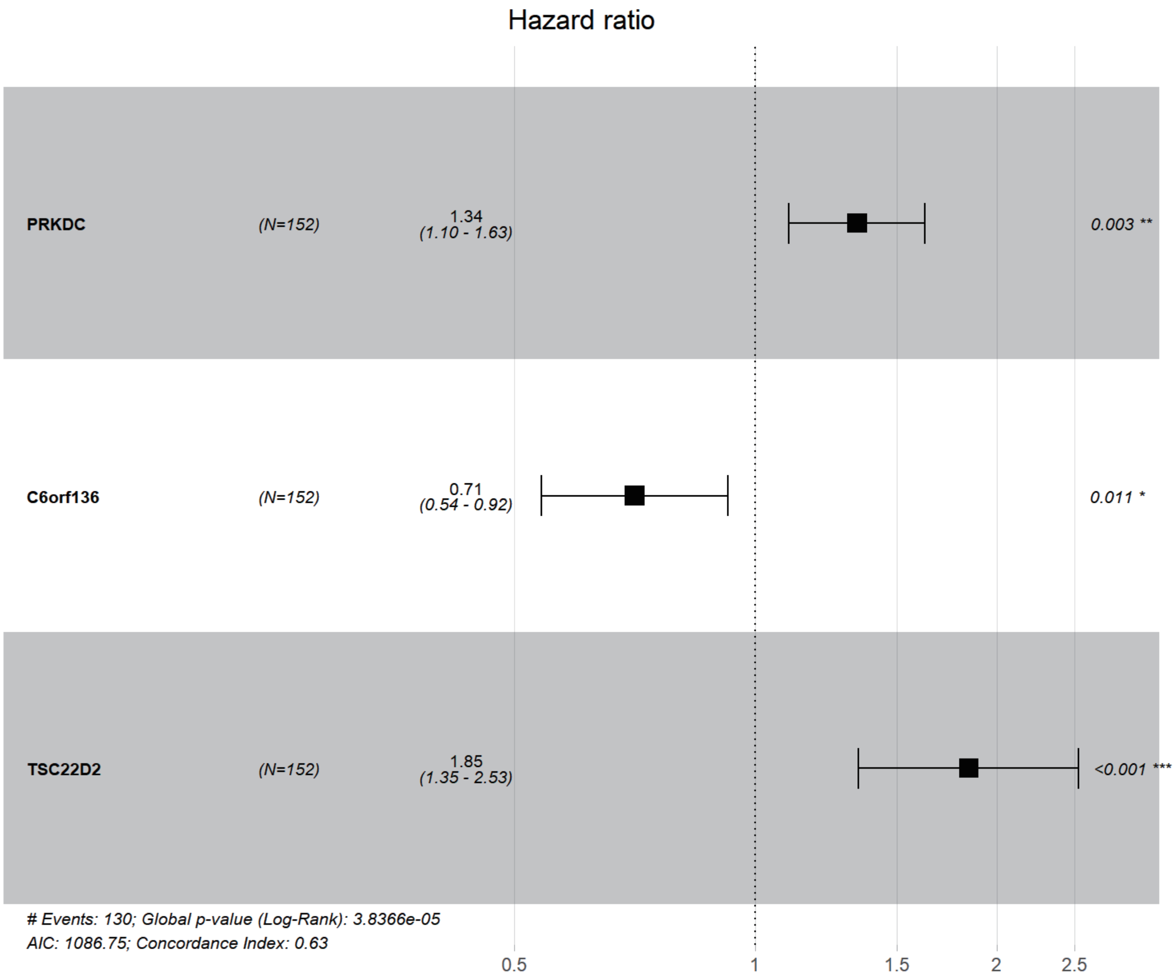

Supplementary Figure 2

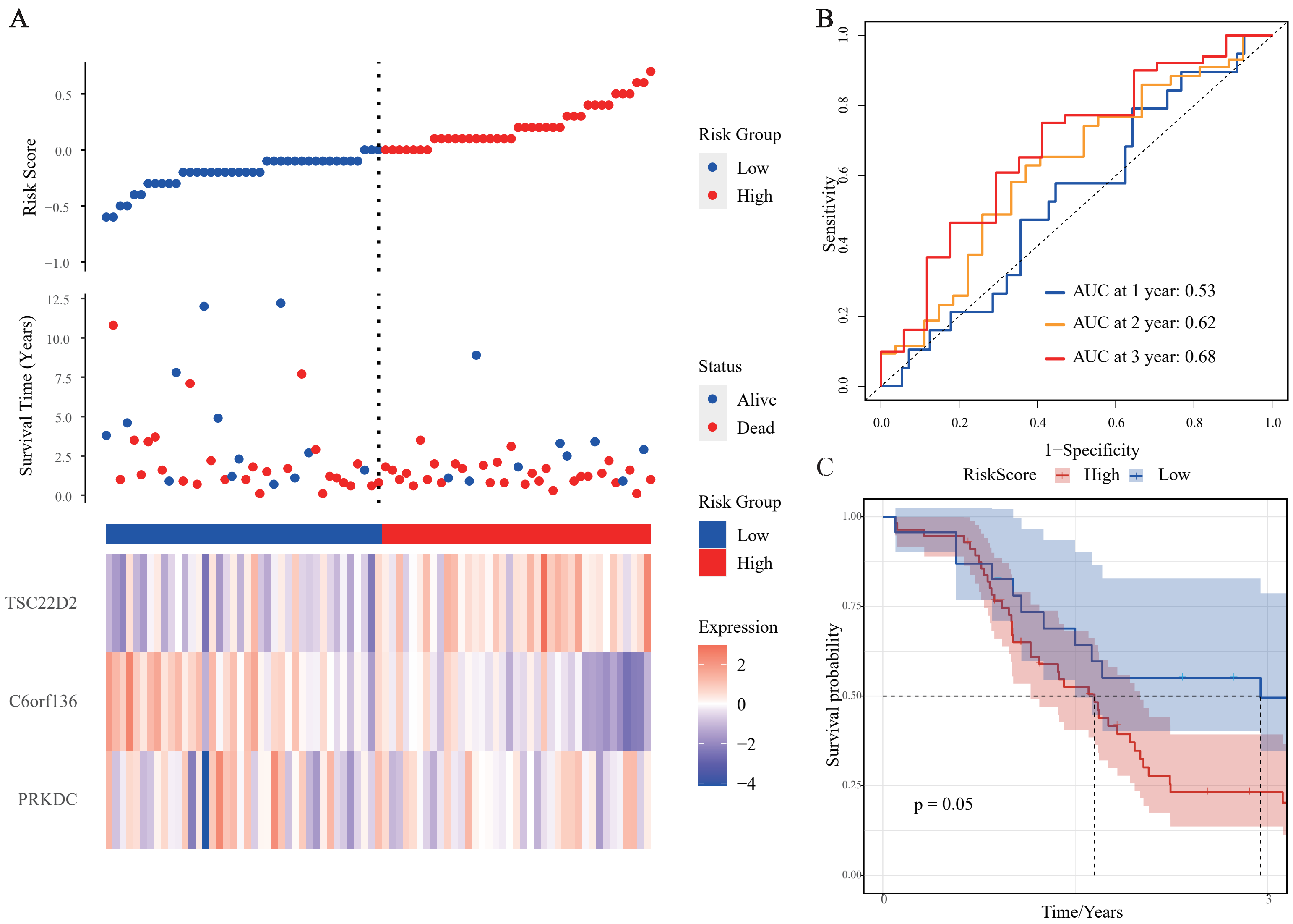

Supplementary Figure 3

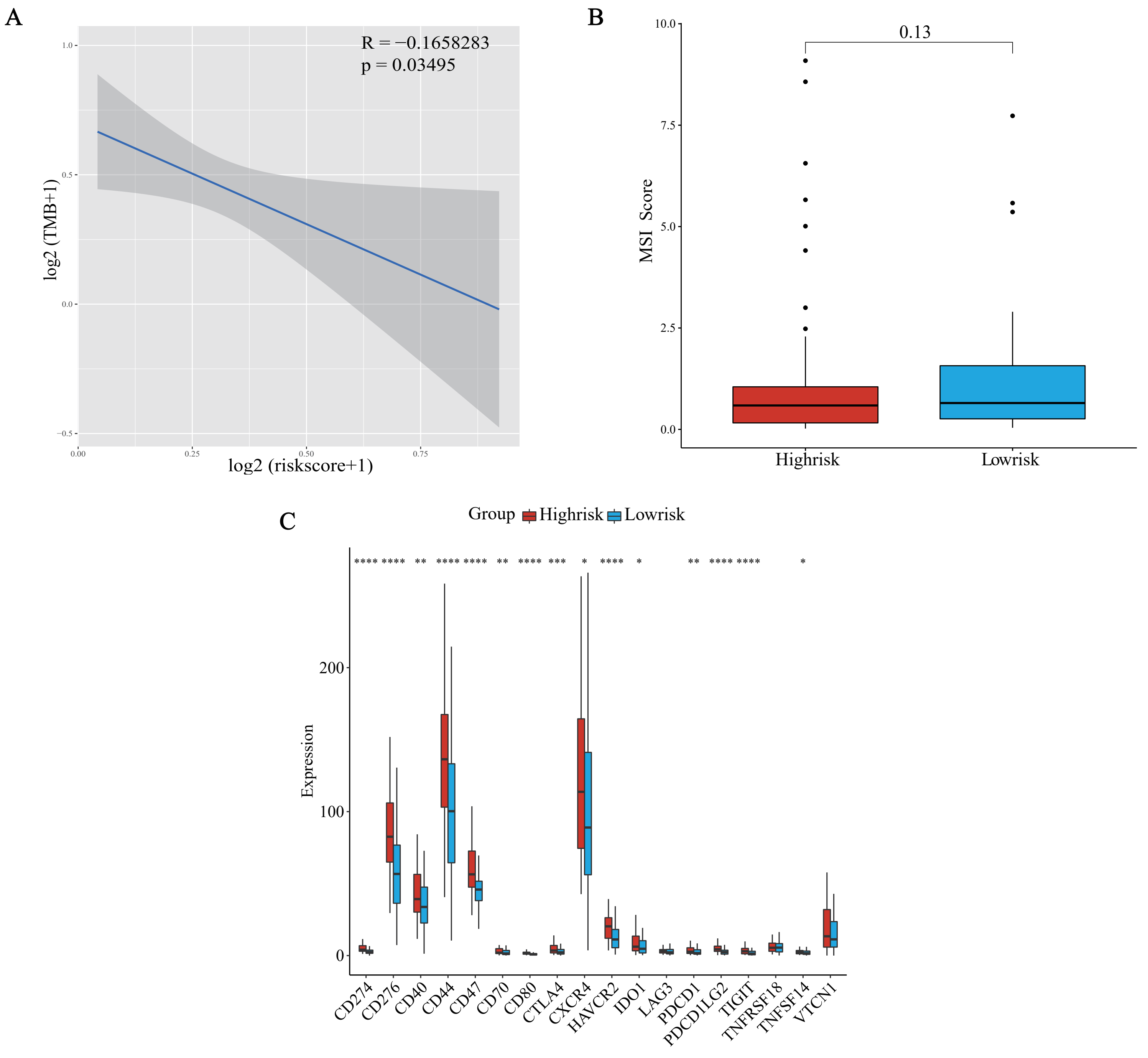

Supplementary Figure 4

A

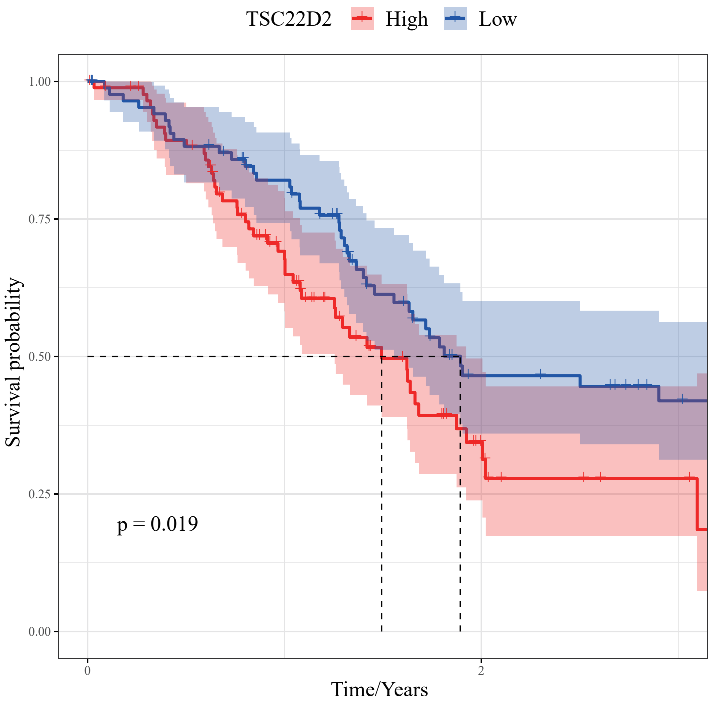

B

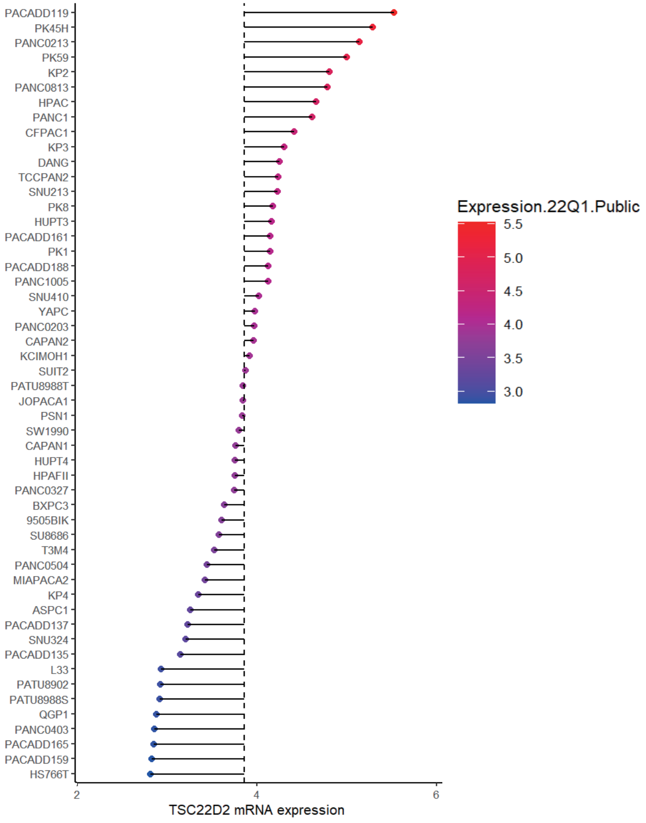

Supplementary Figure S1

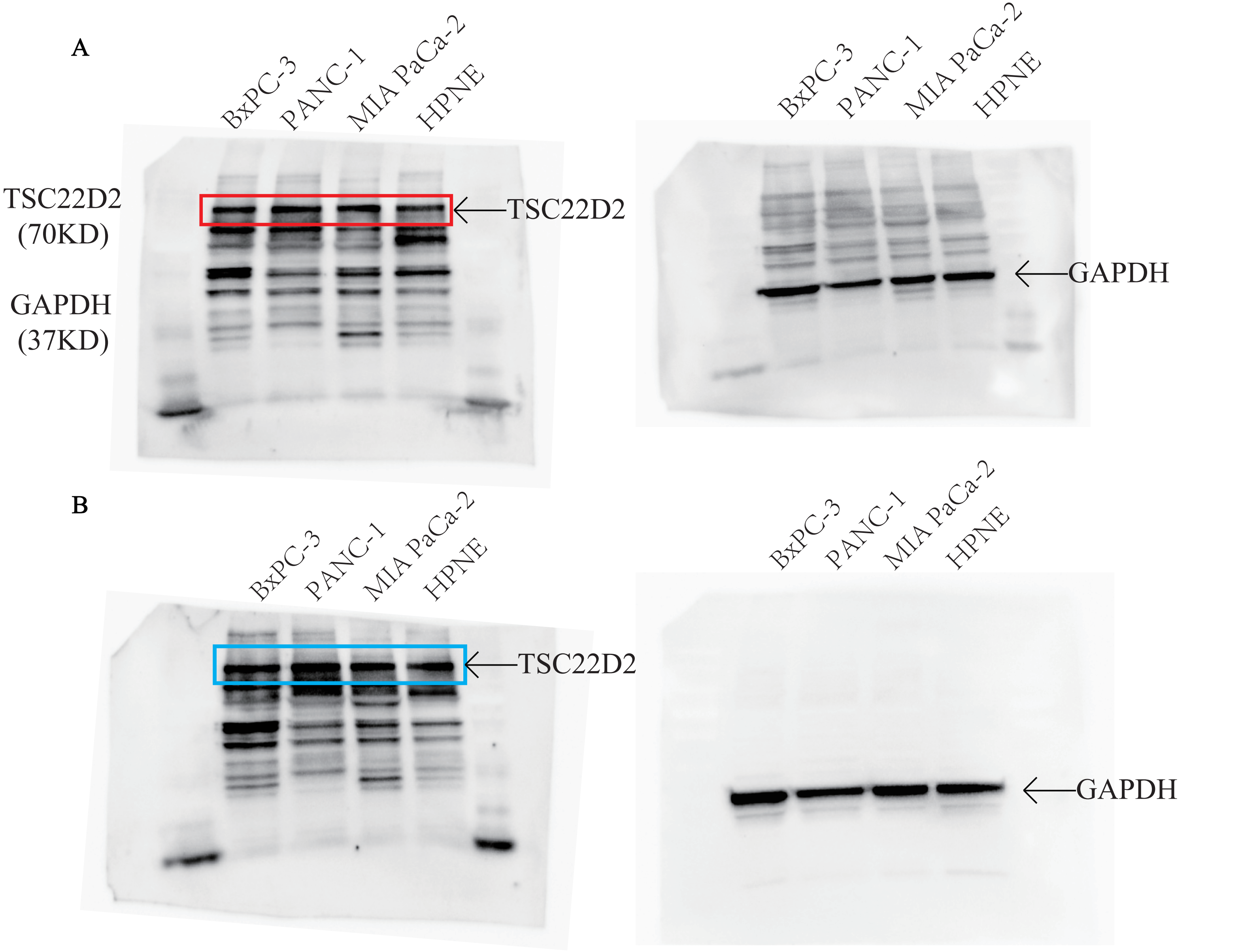

Supplement: Supplementary file 1 — Additional file 1. Supplementary File S1. Cuproptosis-related genes selected by genome-wideCRISPR-Cas9 loss-of-function screens after treatment with elesclomol-copper inOVISE cells. Supplementary Table 1. Differences in clinicopathological characteristics betweenICGC training set and ICGC validation set. Supplementary Figure 1. The hazard ratio of three genes was calculated bymultivariate Cox regression analysis in the prognostic model. Supplementary Figure 2. Evaluation of prognostic model in the GEO testing set. (A)The risk score, survival time, survival status, and 3-gene expression trend in thetesting set (GSE85916, 80 samples). (B) ROC curves for the sensitivity andspecificity of one-, two-, and three-year OS according to the risk score in theGEO-testing set. (C) Kaplan–Meier curve for OS between the high- and low-riskgroups in the GEO-testing set. Supplementary Figure 3. Comparison of the molecular characteristics and immunelandscapes between the risk groups. (A) Correlation between the risk score and TMB.The Spearman correlation coefficients (R) and corresponding p values are shown. (B)The relationship between the risk score and MSI in TCGA-PAAD cohort. (C) Theexpression levels of representative immune checkpoint genes in the high- andlow-risk PAAD patients from the TCGA cohort. * p < 0.05; ** p < 0.01; *** P <0.001; **** P < 0.0001. Supplementary Figure 4. The prognostic value and expression of TSC22D2. (A)Kaplan–Meier curve for OS between the high- and low-TSC22D2 expression groupsin the TCGA cohort. (B) The expression of TSC22D2 in pancreatic cancer cells wasanalyzed in the Cancer Cell Line Encyclopedia database. Supplementary Figure S1. (A-B) Original western blot images of TSC22D2 andGAPDH. [file 12885_2023_10678_MOESM1_ESM.pdf]
